# Brain Morphological Modifications in Congenital and Acquired Auditory Deprivation: A Systematic Review and Coordinate-Based Meta-Analysis

**DOI:** 10.3389/fnins.2022.850245

**Published:** 2022-03-28

**Authors:** Anaïs Grégoire, Naïma Deggouj, Laurence Dricot, Monique Decat, Ron Kupers

**Affiliations:** ^1^Department of ENT, Cliniques Universitaires Saint-Luc, Brussels, Belgium; ^2^Institute of NeuroScience (IoNS), UCLouvain, Brussels, Belgium; ^3^Department of Neuroscience, Panum Institute, University of Copenhagen, Copenhagen, Denmark; ^4^Ecole d’Optométrie, Université de Montréal, Montréal, QC, Canada

**Keywords:** deafness/hearing loss, sign language (SL), neuroplasticity, brain morphology, cochlear implant, MRI, systematic review and meta-analysis

## Abstract

Neuroplasticity following deafness has been widely demonstrated in both humans and animals, but the anatomical substrate of these changes is not yet clear in human brain. However, it is of high importance since hearing loss is a growing problem due to aging population. Moreover, knowing these brain changes could help to understand some disappointing results with cochlear implant, and therefore could improve hearing rehabilitation. A systematic review and a coordinate-based meta-analysis were realized about the morphological brain changes highlighted by MRI in severe to profound hearing loss, congenital and acquired before or after language onset. 25 papers were included in our review, concerning more than 400 deaf subjects, most of them presenting prelingual deafness. The most consistent finding is a volumetric decrease in white matter around bilateral auditory cortex. This change was confirmed by the coordinate-based meta-analysis which shows three converging clusters in this region. The visual areas of deaf children is also significantly impacted, with a decrease of the volume of both gray and white matters. Finally, deafness is responsible of a gray matter increase within the cerebellum, especially at the right side. These results are largely discussed and compared with those from deaf animal models and blind humans, which demonstrate for example a much more consistent gray matter decrease along their respective primary sensory pathway. In human deafness, a lot of other factors than deafness could interact on the brain plasticity. One of the most important is the use of sign language and its age of acquisition, which induce among others changes within the hand motor region and the visual cortex. But other confounding factors exist which have been too little considered in the current literature, such as the etiology of the hearing impairment, the speech-reading ability, the hearing aid use, the frequent associated vestibular dysfunction or neurocognitive impairment. Another important weakness highlighted by this review concern the lack of papers about postlingual deafness, whereas it represents most of the deaf population. Further studies are needed to better understand these issues, and finally try to improve deafness rehabilitation.

## Introduction

### Hearing Loss and Deafness

It is estimated that around 1.5 billion people worldwide experience some degree of hearing loss, which could grow to 2.5 billion by 2050 due to expected population growth and increased longevity (Geneva: World Health Organization, 2021). Although hearing loss can be congenital, affecting two in one thousand newborns, in the vast majority of the cases it is acquired later in life. Hearing impairment is characterized according to severity, ranging from mild to profound forms. The term of deafness is used in case of severe or profound hearing loss, with auditory thresholds greater than 70 dB HL (Hearing Level). Deafness is often classified into prelingual and postlingual, depending on whether its onset occurred before or after learning language, i.e., around the age of four. More than 50% of congenital hearing loss is genetic in origin (e.g., mutation of the connexin 26); other causes are complications at birth or in the neonatal period (birth asphyxia, low birth weight, and severe jaundice), certain infectious diseases (*in utero* infection by cytomegalovirus, rubella, or meningitis), chronic ear infections, ototoxic drugs (antibiotics like aminoglycosides, anti-tumor drugs like cisplatin), exposure to harmful noise levels (in a professional or recreational context), and ageing (Geneva: World Health Organization, 2021). The rate of moderate to severe (disabling) hearing loss increases exponentially with age in all regions of the world, rising from 15.4% among people aged in their 60s, to 58.2% among those aged more than 90 years (Geneva: World Health Organization, 2021). Recent studies on age-related hearing loss highlighted its association with enhanced risk of cognitive decline, depression and social isolation (Lin et al., 2013; Manrique-Huarte et al., 2016; Livingston et al., 2017; Maharani et al., 2019; Griffiths et al., 2020). Care and prevention, such as treatment of ear diseases, vaccination and limitation of the exposure to ototoxic drugs and noise, are essential to limit the negative consequences. The socio-economic impact of hearing loss can be substantial and depends on the severity and the age of onset of the hearing loss. Besides the direct medical costs, there are costs related to special education, vocational rehabilitation, and unemployment or lost productivity (Mohr et al., 2000; Keren et al., 2002; Shield, 2006). For example, Mohr et al. Mohr et al. (2000) estimated that in the United States, severe to profound postlingual hearing loss costs society $ 297.000 over the lifetime of an individual, and more than $ 1 million in case of prelingual deafness. Early intervention can significantly reduce these costs. A study comparing the lifetime societal costs of congenital deafness for a deaf child was estimated at $ 697.500 in case of normal language (due to early intervention), doubling to $ 1.126.300 in case of delayed language (Keren et al., 2002).

**FIGURE 1:**
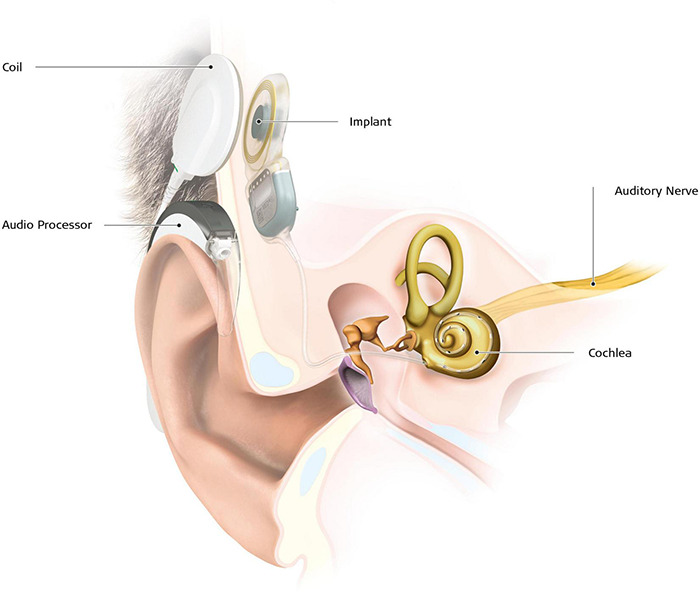
Cochlear implant with its two main components. The external part, which is placed behind the ear, is composed of a microphone, an audio processor, a battery and a coil, kept in place in front of the internal part by a magnet. The internal part is placed during a surgical procedure under general anesthesia. The implant transmits the electrical stimulation to the fibers of the cochlear nerve through electrodes placed in the cochlea (from MED-EL© 2021).

Early access to any type of hearing aids or sign language (SL) is therefore of utmost importance. In case of severe to profound deafness, especially if speech intelligibility with conventional hearing aids is poor (Zwolan et al., 2001), cochlear implant (CI) is the intervention of choice. A CI device consists of an internal part, with an electrode array placed directly in the cochlea, delivering electric stimulation to the branches of the auditory nerve, and an external part composed of a microphone and a speech processor (Figure 1). Most of the time, the CI allows oral language development in prelingual deafness, and functional hearing in postlingual deafness (Fulcher et al., 2012; Gaylor et al., 2013). According to estimates based on manufacturers’ voluntary reports of registered devices to the United States Food and Drug Administration, 183.000 subjects have been implanted in the United States in 2019, of which a little more than a third were children. In case of prelingual deafness, CI implantation has to take place at least before the end of the language sensitive period to enable the normal development of auditory pathways (Ponton et al., 1996), and even before 9 months of age for optimum language development (Dettman et al., 2021). Despite significant improvement in mean speech scores after implantation (Gaylor et al., 2013), the variability of the outcomes is important, with speech discrimination in quiet conditions ranging from 0 to 100% (Lazard et al., 2012). The rate of poor performers varies from 10 to 50% depending on the definition used (Moberly et al., 2016). The reasons for poor CI success rates are unclear. For instance, a large multicentric study on 2,251 CI patients revealed that residual hearing, percentage of active electrodes, use of hearing aids during the period of profound hearing loss, and duration of moderate hearing loss only explain 20% of the variance (Lazard et al., 2012). Other studies reported that the remaining spiral ganglion cell count, representing the integrity of the peripheral auditory pathway, also does not explain the variance in speech perception (Fayad et al., 1991; Blamey et al., 1996). Thus, other factors must affect performance with a CI, such as the integrity of the central auditory pathways or already established brain plasticity (Sharma and Glick, 2016). Indeed, there is now ample evidence from both animal and human studies that the deprived auditory cortical areas process other sensory signals, like vision and touch (Petitto et al., 2000; Finney et al., 2001; Allman et al., 2009; Campbell and Sharma, 2014), a process referred to as cross-modal plasticity. Although cortical plasticity following loss of vision has been described in detail [see Kupers and Ptito (2014); for a review], there is a relative sparsity in studies on brain plastic changes following auditory loss. There is some evidence that structural and functional brain changes can impact rehabilitation with a CI (Lee et al., 2007; Lazard et al., 2013; Tan et al., 2015; Feng et al., 2018; Han et al., 2019; Wang et al., 2019; Sun et al., 2021). Furthering our understanding of brain plasticity and its structural substrate in deafness is therefore of utmost importance to improve therapeutic outcomes of CI therapy.

The aim of this systematic review and coordinate-based meta-analysis is to summarize the current knowledge on gray and white matter changes as revealed by structural MRI in subjects with severe to profound hearing loss. Plastic changes in deaf humans are compared with those in deaf animals and in human blind subjects. We end with some perspectives and further possible directions of investigation.

### Animal Model of Deafness

Several animal models of deafness have been used to study neuroplastic changes following loss of audition. The most widely used animal model is the congenitally deaf white cat (Heid et al., 1998). These animals suffer from a genetic disease which means that 70 percent of them are born deaf. The auditory system of the cat is similar to that of humans in terms of auditory fields, gyrification and acoustic functions, making the deaf white cat a suitable model for studying congenital deafness (Kral and Lomber, 2015). Other animal models include transgenic mice (Kozel et al., 1998; Hunt et al., 2006), congenitally deaf dogs (Niparko and Finger, 1997), animals deafened at different ages by administration of ototoxic drugs (Hardie and Shepherd, 1999; Meredith and Allman, 2012), and gerbils deafened by aspiration of the content of the cochlea (Kitzes and Semple, 1985; Moore and Kitzes, 1985).

The modifications of the auditory pathways in animal models of deafness are discussed below according to structural or functional criteria.

### Structural Changes in Animal Model of Deafness

**FIGURE 2:**
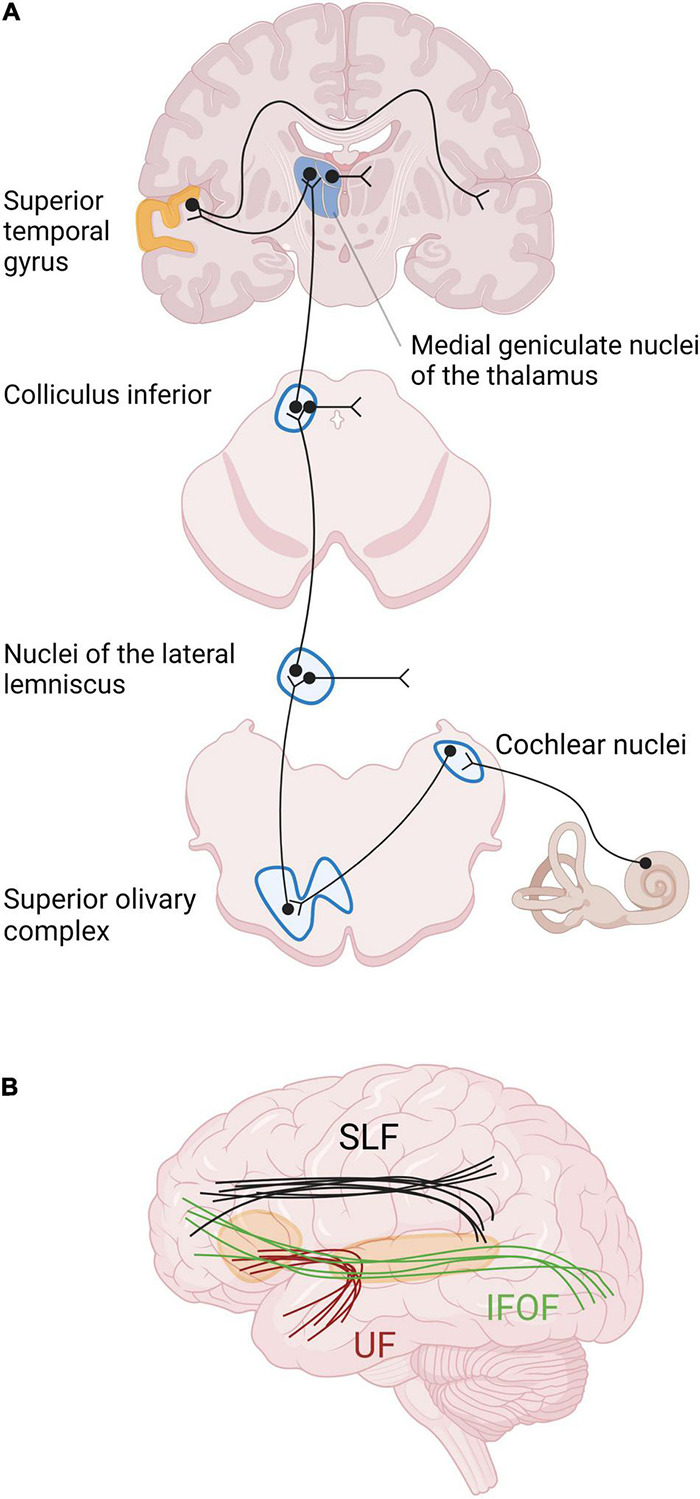
Primary auditory pathway and main white matter tracts involved in auditory processing and language. **(A)** The relays of the auditory pathway are the cochlear nuclei, the superior olivary complex, the nuclei of the lateral lemniscus for some neurons, the inferior colliculus and the medial geniculate nucleus of the thalamus. The 8th cranial nerve finally reaches the auditory cortex in the superior temporal gyrus. The major part of the fibers from the cochlea cross the medial line at different levels of the neuraxis and arrive in contralateral auditory cortex. **(B)** The main intrahemispheric white matter bundles involved in auditory processing and language are the superior longitudinal fasciculus (SLF), the inferior fronto-occipital fasciculus (IFOF), and the uncinate fasciculus (UF), schematically represented here (Maffei et al., 2015).

Structural changes have been described at the different relays of the auditory pathway (Figure 2). Studies in neonatally deaf animals revealed that the cochlear nuclei, which form the first relay of the auditory pathway, are decreased in global volume, cell body size, and neuronal density (Hultcrantz et al., 1991; Niparko and Finger, 1997; Butler and Lomber, 2013). The magnitude of these changes was correlated with the duration of hearing impairment, and thus also with age (Hardie and Shepherd, 1999). In contrast, no such changes were shown in animals with late-onset deafness (Tierney et al., 1997; Stakhovskaya et al., 2008). The next relay of the auditory pathway is the superior olivary complex, composed of three main nuclei surrounded by smaller periolivary nuclei. In animals with early-onset of deafness, the size and the number of neurons within the main olivary nuclei are decreased, and the tonotopy is disrupted [see Butler and Lomber (2013) for a review]. More rostrally, the volume of the inferior colliculi and their constituent neurons is decreased in animals with early-onset deafness, despite the fact that the total number of neurons remains globally stable [see Butler and Lomber (2013) for a review]. The thalamus has been very little studied in animal models of deafness, probably due to difficulty accessing the thalamic structures (Butler and Lomber, 2013). However, a study of thalamic projections to the primary auditory cortex A1 in neonatally deafened cats, using a retrograde tracer, showed that the labeling in the ventral division of the medial geniculate body did not differ between hearing and deafened animals (Stanton and Harrison, 2000).

**FIGURE 3:**
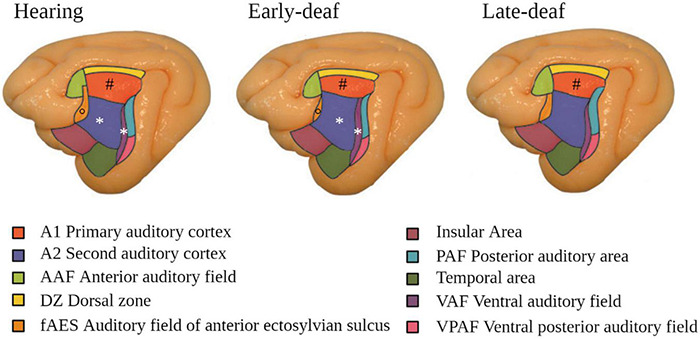
Modifications in the auditory cortex in early- and late-deaf cats. Cytoarchitectonic study showing that A1 is reduced in both early- and late-deaf cats (#) compared to hearing cats. Additionally, in early-deaf cats, A2 and VAF are larger (*), whereas fAES is smaller compared to normal hearing cats (°). Data adapted from Wong et al. (2014) with permission of the author and publisher.

At the level of the auditory cortex, studies in deaf cats showed an overall trend to a decrease in size, in particular in early-deaf cats (Lomber et al., 2019). In both early- and late-deaf cats, this global decrease was driven by a reduced volume of the primary auditory cortex (PAC), despite a conservation of its laminar structure (Hartmann et al., 1997). The secondary auditory areas also showed changes in size and of their borders with adjacent auditory, visual or somatosensory regions, which depended on the onset of deafness. For example, in early deaf cats compared to hearing cats, A2 was larger and the auditory field of the anterior ectosylvian sulcus (FAES) was smaller, whereas in late-deaf cats the posterior auditory field (PAF) was expanded (Figure 3) (Wong et al., 2014; Chabot et al., 2015). In congenitally deaf mice, the primary visual cortex was enlarged (Hunt et al., 2006).

Berger and co-workers compared the laminar organization of the auditory cortex in congenitally deaf and hearing cats. Although the auditory cortex preserved its six-layered cortical structure, the granular (IV) and infragranular (V–VI) layers were thinner in both A1 and the dorsal zone (DZ), which is part of the secondary auditory cortex (Berger et al., 2017). The thinner infra-granular layers were explained by a combination of factors such as a reduction of the cell soma size, smaller dendritic trees, and reduced number and size of synaptic spines (Kral and Sharma, 2012; Berger et al., 2017). In contrast, there was no change in the global size of the supragranular layers (I to III), despite an increase in dendritic branching in A1 and the FAES in long-term neonatally deaf cats (Clemo et al., 2016, 2017).

### Functional Changes in Animal Model of Deafness

Improved visual and tactile functions in both early- and late-deaf animals are well-documented (Rebillard and Pujol, 1977; Allman et al., 2009; Lomber et al., 2010; Meredith and Lomber, 2011). For example, congenitally deaf cats show better visual localization in the peripheral field and lower visual movement detection thresholds (Lomber et al., 2010). There is evidence that DZ and PAF play a significant role in these compensatory plastic changes since inactivating these areas by local cooling makes thresholds return to normal (Lomber et al., 2010). Alterations in the response specificity of primary auditory cortical neurons have also been described (Hunt et al., 2006; Meredith and Lomber, 2011; Meredith et al., 2012): whereas more than 90% of neurons in A1 of normal hearing animals respond exclusively to auditory input, 68% of these neurons responded to unimodal somatosensory stimuli in congenitally deaf mice (Hunt et al., 2006). Plastic functional changes also take place in case of partial acquired hearing loss. For instance, Meredith et al. (2012) showed that the number of unimodal auditory neurons decreased from 65 to 31%, whereas neurons responding to different sensory modalities increased from 34 to 68% in partially hearing-impaired ferrets.

Local field potential recordings after intracochlear electrical stimulation showed that synaptic activity is preserved in the supragranular layers of the PAC in deaf cats, but is decreased in the infragranular layers (Klinke et al., 1999; Kral et al., 2000). This is in line with results from cytoarchitectural studies described above (Berger et al., 2017). The supragranular layers process bottom-up sensory-input, whereas the infragranular layers are the source of the outputs to the thalamic nuclei, the auditory midbrain and other brainstem structures (Schofield, 2009; Znamenskiy and Zador, 2013; Mellott et al., 2014). These layer-specific responses to electric cochlear stimulation suggest that the connections between the supra- and infragranular layers do not mature normally in congenitally deaf animals (Lomber et al., 2019).

Different mechanisms have been proposed to explain the above described functional and structural cortical changes. A first mechanism is the strengthening of existing cortico-cortical connections by the development of new synapses in the core and belt auditory cortex, both in early and late-onset deafness (Kok et al., 2014; Wong et al., 2015; Barone et al., 2016). For example, Wong et al. (2015) showed increased connections from visual and somatosensory areas to the anterior auditory field (AAF), as well as decreased connections from other auditory areas to AAF and PAF (Yusuf et al., 2021). These modifications were more pronounced in early-deaf compared to late-deaf cats. Together with A1, the AAF forms the core of the auditory cortex. Other authors reported an increase in projection strength from secondary visual areas (area 7 and region of the suprasylvian sulcus) to the secondary auditory area DZ in both early and late deaf cats (Kok et al., 2014; Barone et al., 2016). A second possible mechanism underlying cortical plasticity is the formation of new cortico-cortical connections. Although one study reported new projections to DZ from visual areas 19/20 and the multimodal areas of the anterior ectosylvian sulcus and the orbito-frontal region (Barone et al., 2013), there are no other reports in support of such a mechanism (Cardin et al., 2020). Finally, it has been suggested that cross-modal plastic changes are mediated by changes at the level of the brainstem (Allman et al., 2009; Butler et al., 2017). Although inputs from the somatosensory system to the dorsal cochlear nucleus and the inferior colliculus have been demonstrated in normal hearing animals (Aitkin et al., 1981; Shore et al., 2000; Kanold and Young, 2001; Shore and Zhou, 2006), these are enhanced after hearing loss, and their response thresholds are decreased (Shore et al., 2008; Meredith and Allman, 2012).

To summarize, there is ample evidence for a reduction of the size or the number of neurons at different levels of the auditory pathways and in the PAC, especially in case of early deafness. However, the global pattern of thalamo-cortical and cortico-cortical connections seems mainly preserved, with some strengthening but very few *de novo* connections. The responsiveness of PAC neurons to non-auditory sensory inputs may underlie the improved tactile and visual skills in deaf animals.

### Structural Brain Modifications in Human Deafness

Enhancement of non-auditory sensory skills has also been reported in human deafness. For example, improved visual functions have been described in congenitally deaf individuals, including distinguishing emotional facial expressions and local facial features, peripheral visual field tasks and attention to the peripheral visual field (Neville and Lawson, 1987a; McCullough and Emmorey, 1997; Arnold and Murray, 1998; Bavelier et al., 2000; Hauthal et al., 2013; Almeida et al., 2015). Activation of auditory areas has been demonstrated in response to somatosensory or visual stimuli, including sign language, in congenitally and acquired deaf humans, even in those presenting only a moderate hearing loss (Petitto et al., 2000; Finney et al., 2001, 2003; Levänen and Hamdorf, 2001; Schürmann et al., 2006; Campbell and Sharma, 2014). In line with results obtained in congenital blindness, the deprived auditory cortex becomes also involved in higher-order cognitive functions such as working memory and language (Buchsbaum et al., 2015; Ding et al., 2015; Cardin et al., 2020). In accordance with results from animal models of deafness (Diamond and Weinberger, 1984; Kral et al., 2003; Lomber et al., 2010; Meredith et al., 2011) and blindness (Rauschecker and Korte, 1993; Yaka et al., 1999), the higher-order auditory areas seem to have a higher capacity for plastic reorganization than the primary area (Kral, 2007; Butler and Lomber, 2013; Cardin et al., 2013, 2016, 2020). However, the structural basis underlying this functional plasticity remains an issue of debate (Lomber et al., 2019). A post-mortem histological study in subjects who suffered from profound deafness revealed that the cell bodies in the cochlear nuclei were larger at the side of the least affected ear (Chao et al., 2002).

In order to improve our understanding of the structural correlates of deafness associated functional plasticity, we conducted a systematic review and coordinate-based meta-analysis of the anatomical MRI studies conducted in subjects with severe to profound hearing loss.

## Methods

### Search Strategy and Paper Selection

We performed a systematic review of the literature on studies published in English language peer-reviewed journals and comparing gray matter (GM)/white matter (WM) volume, cortical thickness, and cortical curvature between severe to profound deaf and normal hearing subjects. We followed the PRISMA guidelines (Page et al., 2021). The search was conducted in June 2020, using PubMed and Embase, with the MeSH search terms “magnetic resonance imaging” AND “deafness”, the Emtree search terms “nuclear magnetic resonance imaging” AND “hearing impairment” OR “morphometry” AND “hearing impairment” and the key search terms “magnetic resonance imaging,” “MRI,” “morphometry,” “cortex volume,” “deaf,” and “deafness.” We screened all titles and abstracts and excluded studies of unilateral hearing loss, central hearing loss, tinnitus, and studies without a normal hearing control group. We built a chart collecting for each study the data about (1) meta-study information (e.g., authors and year of publication), (2) characteristics of the population (e.g., age, deafness and language characteristics, use of hearing aids, and handedness), (3) neuroimaging methods (e.g., region of interest or whole brain analysis, manual or voxel-based morphometry), technical information related to MRI and image acquisition (e.g., magnetic field strength, pulse sequences, and image resolution), (4) and significant results, if possible corrected for multiple comparisons. We encoded significant differences of global or regional brain volumes, GM and WM volumes, cortical thickness and curvature, specifying the hemisphere affected. When available, the MNI (Montreal Neurological Institute) or Talairach stereotactic coordinates were noted and reported on MRI for visualization. Since the size of the brain area affected by the change in volume or thickness was only rarely reported, it was not taken into account. This review has not been registered.

We classified the papers using the Oxford Center for Evidence-based Medicine (OCEBM) levels of evidence (Howick et al., 2011). Level 1 corresponds to systematic review, level 2 to randomized trials, level 3 to cohort studies and non-randomized controlled trial, and level 4 to case-series and case-control studies. We evaluated the quality of the included studies using the Newcastle-Ottawa quality assessment Scale (NOS) (Wells et al., 2000). In this scale, each study is judged on eight items, categorized into three groups: the selection of the study groups; the comparability of the groups, and the ascertainment of the exposure. The highest quality studies are awarded up to nine stars. The NOS is the most widely used instrument to assess quality of case-control and cohort studies (Luchini et al., 2017). However, some authors complained about a low inter-rater agreement (Hartling et al., 2013).

### Activation Likelihood Estimation Meta-Analysis

We also performed an Activation Likelihood Estimation (ALE) meta-analysis of the literature of structural changes in deafness, based on the reported stereotactic coordinates. ALE was initially developed for meta-analysis of functional data (Turkeltaub et al., 2002), but the current version, GingerALE Version 3.0.2, has been adapted for brain structural analysis as well (Eickhoff et al., 2009). This technique gives a probabilistic localization of overlapping foci from different studies. The foci are transformed in spatial probability distributions centered at the given coordinates, with a width based on empirical estimates of spatial uncertainty due to the inter-subject and inter-template variability. It is important to note that this analysis does not take into account the extent of the reported brain modifications. The ALE results are finally compared with the null-distribution of the random spatial association between studies, resulting in a random-effect inference (Eickhoff et al., 2012).

To be included, the studies have to use Voxel-Based Morphometry (VBM), to provide the coordinates of the peaks in MNI or Talairach space, and to give significant results after correction for multiple comparisons. All the coordinates were transformed in MNI space, using SPM in GingerALE. Two datasets were built, the first one with the coordinates of the peaks where deaf subjects showed greater volume than the normal hearing control subjects, and the other one with the peaks where deaf subjects showed lower volume than their controls. For the paper from Olulade et al. (2014), which compared four subgroups depending on the level of hearing (normal or profoundly impaired) and the type of language (oral or SL), we chose to only include the coordinates of peaks from the comparison deaf versus normal hearing SL users in order to avoid potential changes related to SL use. As recommended by Eickhoff et al. (2012), we applied to output images thresholds of *p* < 0,01 (cluster forming threshold) and 0,05 for cluster-level family-wise-error (FWE) with 1,000 thresholding permutations.

## Results

**FIGURE 4:**
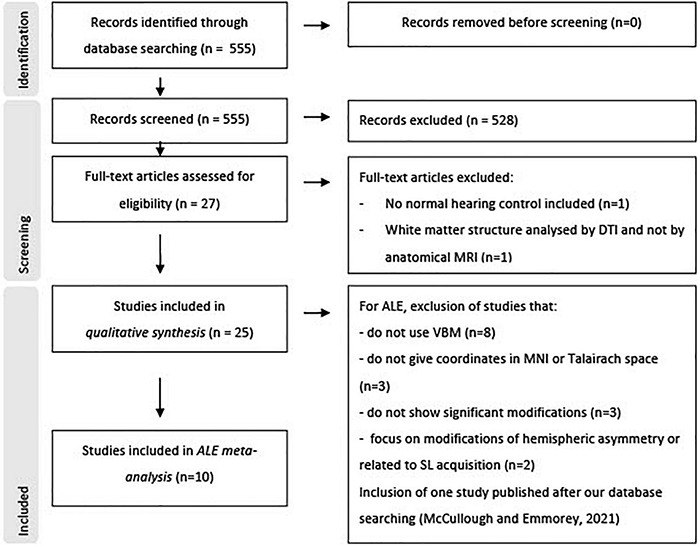
Study flowchart according to PRISMA 2020.

A total of 555 studies were identified and screened from all database searching (see Figure 4 for flowchart PRISMA). Of these, 528 papers were excluded based on their title or abstract, mostly because their subjects did not present severe or profound bilateral hearing loss, or no anatomical MRI was included, or for a lack of a normal hearing control group. From the 27 remaining papers, 25 were finally included in the narrative systematic review, and nine in the coordinate-based meta-analysis. However, a recent tenth study was included in the meta-analysis to enhance statistical power (McCullough and Emmorey, 2021). All papers concerned case-control studies with an OCEBM level of evidence of 4 and are of good quality according to the Newcastle-Ottawa quality assessment Scale.

### Demographics and Global Overview

In total, 427 deaf individuals and 539 normal hearing controls were included (see Supplementary Table 1 for demographic characteristics). Of these, 110 were deaf children, 184 normal hearing children, 317 deaf adults, and 355 normal hearing adults. Several studies shared partially or totally the same population, but they focused on different regions of interest (ROIs) or used various methods (Supplementary Table 1). The age of the participants ranged from a few months until 70 years (mean age 24.5 years ± 1.7 SD). Seven studies focused on a pediatric population. Of these, two studies included toddlers from 8 to 38 months (Smith et al., 2011; Feng et al., 2018), one included children from 1 to 9 years (Shiohama et al., 2019), one included children from 5 to 14 years (Shi et al., 2016); three studies used the same population of children and adolescents from 10 to 18 years (Li J. et al., 2012; Li et al., 2013, 2015). Thirteen studies included participants with profound deafness (hearing loss over 90 dB), five included participants with severe to profound deafness (>70 dB). Seven studies had a mixture of patients with moderate hearing loss and severe to profound deafness (>50 dB HL) (Emmorey et al., 2003; Allen et al., 2008, 2013; Smith et al., 2011; Feng et al., 2018; Qi et al., 2019; Shiohama et al., 2019). For the study by Shiohama et al. (2019), we only took into account the group of children with severe to profound hearing loss, excluding those with mild to moderate hearing loss. In 24 out of 25 publications, only congenitally or prelingually deaf subjects were studied, most of them using SL as main language. Only one study included both pre- and postlingually deaf subjects (Kim et al., 2014). In most studies, deaf and hearing groups were matched for age, sex and handedness. Most studies excluded left-handed subjects, which increases population homogeneity but may induce selection bias. Cases presenting with additional neurological disease and brain malformation were excluded. Very little information is provided about the exact etiology of deafness (see Supplementary Table 1). The deaf and control groups are difficult to match in terms of level of education, since congenitally deaf people often do not have access to traditional education. However, some studies matched their groups for IQ (Olulade et al., 2014; Kumar and Mishra, 2018), linguistic proficiency (Penhune et al., 2003) or socio-economic status (Feng et al., 2018). Supplementary Table 1 provides information about the demographic and main findings of the included papers.

### Changes in Gray Matter

**FIGURE 5:**
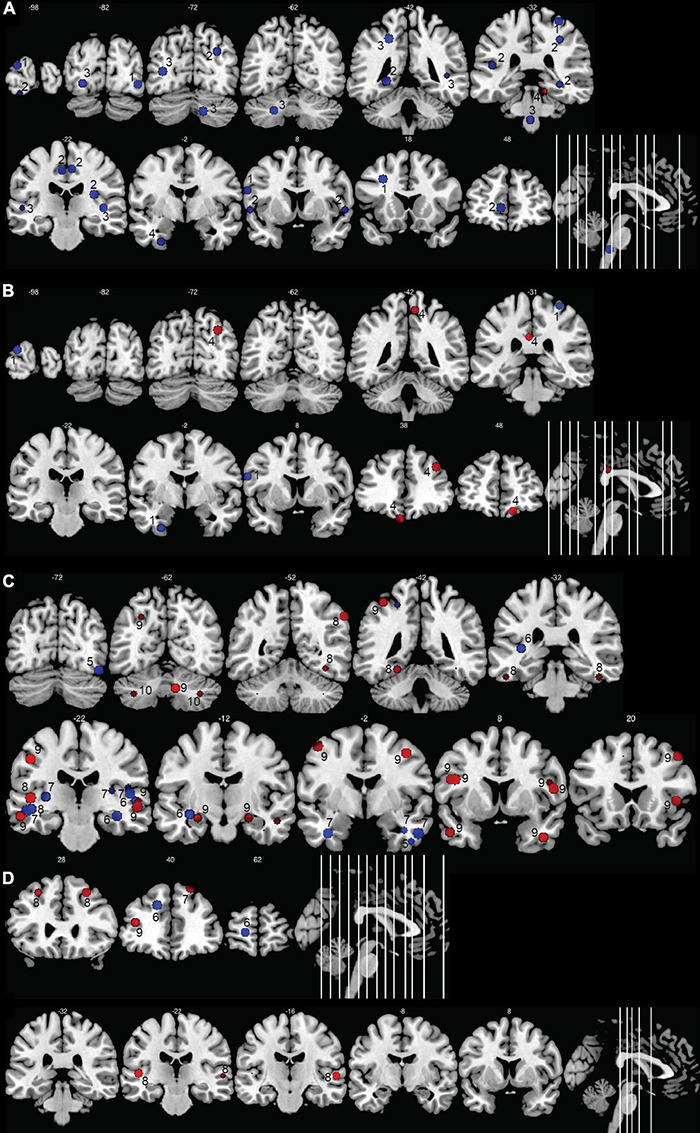
Morphometric changes in the human deaf brain. Increases in GM or WM volume in the deaf compared to hearing controls are shown in red, decreases in blue. **(A)** GM and WM volume in deaf children; **(B)** cortical thickness in deaf children; **(C)** GM and WM volume in deaf adults; **(D)** cortical thickness in deaf adults. The image shows the peak (or center) of the areas with modifications in volume or cortical thickness; cluster volumes are not indicated. Some spheres appear smaller because they are shown on slices which are not positioned at the center of the spheres. Right hemisphere is shown on the right of the images. The numbers on top of the slices show the *y*-coordinates of the coronal slices in MNI space. The numbers inside the slices correspond to the studies from which the coordinates are taken. 1: Li J. et al. (2012); 2: Feng et al. (2018); 3: Smith et al. (2011); 4: Li et al. (2013); 5: Qi et al. (2019); 6: Kim et al. (2009); 7: Olulade et al. (2014); 8: Kumar and Mishra (2018); 9: Leporé et al. (2010a); 10: Hribar et al. (2014).

Figure 5 summarizes the significant brain modifications together with their stereotactic coordinates. The main GM and cortical thickness modifications were found in the visual cortex, extending from the occipital lobe to the fusiform gyrus of the temporal lobe, especially in the pediatric deaf population. Decrease of GM volume or in cortical thickness in deaf babies and children were found in right or left superior occipital gyri (Li J. et al., 2012; Feng et al., 2018), left middle and inferior occipital gyri (Shiohama et al., 2019), left lingual gyrus (Feng et al., 2018), and left fusiform gyrus (Li J. et al., 2012). Three other studies failed to detect GM changes among these visual areas in deaf children (Smith et al., 2011; Li et al., 2015; Shi et al., 2016). GM changes in the visual areas were more inconsistent in the adult deaf population, with half of the studies describing an increase or a decrease in GM. For example, a study of nine prelingually deaf adults, focusing on purported changes in the early visual cortex, did not find any differences in this area (Fine et al., 2005), whereas other studies reported a GM volume increase in the calcarine sulcus (Allen et al., 2013) or fusiform gyrus (Kumar and Mishra, 2018). Still others reported a GM volume reduction in both fusiform gyri (Olulade et al., 2014; Qi et al., 2019) and in the right middle occipital gyrus (Qi et al., 2019). Finally, one study reported a decrease in cortical thickness in the calcarine sulcus of prelingually deaf adults (Smittenaar et al., 2016). The age of SL acquisition may have an influence on the GM volume in the visual cortex. Pénicaud et al. (2013) showed that GM volume is decreased in left primary (V1) and secondary (V2) visual areas, and also in the left dorsal visual association areas (V3a/V7) in late SL learners (acquisition between 11 and 14 years), whereas it is increased in deaf adults with SL acquisition before 3 years old. These authors did not find differences between deaf and hearing subjects when all deaf subjects were analyzed together.

Modifications of GM in the temporal lobe were present in only five out of 19 studies. In deaf babies, one study reported a GM volume increase in the anterior part of Heschl’s gyrus (HG) (Smith et al., 2011); in deaf adults, GM increases were found in bilateral superior temporal gyrus (STG) (Leporé et al., 2010a) or exclusively in the right STG extending posteriorly to the planum temporale (Emmorey et al., 2003), and in inferior temporal gyrus (Kumar and Mishra, 2018). In contrast, one study in deaf babies reported a reduction in GM density in bilateral STG and HG (Feng et al., 2018).

There are also reports of GM changes outside the occipital and temporal brain areas. Three studies in deaf adults reported increased GM volume in the bilateral (Allen et al., 2013; Kumar and Mishra, 2018) or left (Leporé et al., 2010a) inferior frontal gyrus, a brain area which is involved in language production. One study showed a reduction of GM cortical thickness of the left middle frontal gyrus in adolescents (Li et al., 2013); three others reported an increase of GM volume in adults either in both middle frontal gyri (Kumar and Mishra, 2018) or only at the right side (Leporé et al., 2010a; Olulade et al., 2014). One study in deaf babies reported a decrease in GM volume in the right supramarginal gyrus, an area which is part of the somatosensory association cortex (Feng et al., 2018). Three studies reported a GM volume increase in the precentral gyrus, more precisely within the left motor hand region (Leporé et al., 2010a; Li J. et al., 2012; Kumar and Mishra, 2018), likely due to the hand movements during SL production. The use of SL was also associated with a GM increase in different parts of the frontal gyri, especially the bilateral middle frontal, right medial frontal and right inferior frontal gyri (Olulade et al., 2014).

The GM modifications were also found in the precuneus, which is part of the parietal lobe and that plays a role, among others, in the integration of multisensory information. The modifications varied with age, deaf babies showing a GM decrease in the left precuneus (Feng et al., 2018), and deaf adolescents a cortical thickness increase of the right precuneus (Li et al., 2013). The use of SL was associated with a GM increase in the right precuneus (Olulade et al., 2014). Contradictory findings were also reported for the insula, with some studies reporting an increased GM volume in left posterior insula in congenitally deaf adults compared to hearing signers and non-signers (Allen et al., 2008), and other studies reporting a GM decrease in bilateral insula in deaf compared to hearing signers (Olulade et al., 2014). The GM decrease in the right insula seemed related to the use of SL in the congenitally deaf, rather than to deafness *per se* (Olulade et al., 2014). The limbic lobe was affected, with two studies describing a GM decrease in the right cingulate gyrus in deaf babies (Feng et al., 2018) and deaf adults (Olulade et al., 2014), and one study reporting an increase in cortical thickness in the left posterior cingulate gyrus in deaf adolescents (Li et al., 2013). Again, the use of SL, and not deafness, seemed to account for the GM increase in the left cingulate gyrus (Olulade et al., 2014).

**FIGURE 6:**
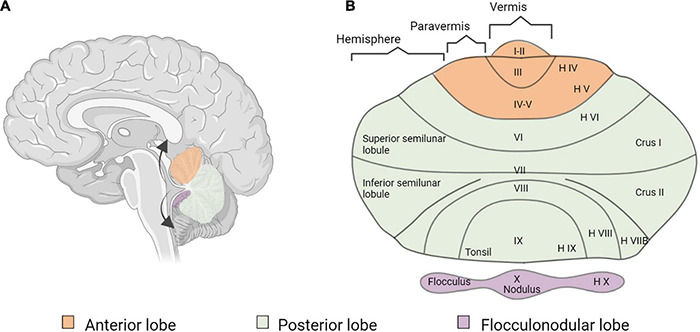
Cerebellar anatomy. **(A)** Sagittal section of the human brain and cerebellum. The arrows show the way the cerebellum is unfolded. **(B)** Flatmap representation of the unfolded cerebellum, using both the classification of Larsell (1952) and the classical nomenclature of the human cerebellum (Malacarne, 1791; Reil, 1808; Burdach, 1829).

Several studies reported increases in GM volume in the cerebellum, either at the right side (Leporé et al., 2010a; Li et al., 2013; Kumar and Mishra, 2018), but sometimes also bilaterally (Hribar et al., 2014) (see Figure 6 for cerebellar anatomy). More specifically, these GM increases occurred in the crus I and II, also called superior and inferior semi-lunar lobules (Hribar et al., 2014; Kumar and Mishra, 2018), in right lobules IX and X (Leporé et al., 2010a) and in right lobules IV and V (Li et al., 2013). Interestingly, this GM increase was less pronounced in participants with long-term use of hearing aids (Li et al., 2013). One study in deaf adults reported a GM decrease in the left crus II and in left lobule VIII (Olulade et al., 2014).

Finally, Amaral et al. (2016) showed that the right thalamus, right lateral geniculate nucleus and right inferior colliculus are larger than their left counterparts in congenitally deaf subjects, suggesting that these subcortical structures participate in the rerouting of visual information to the right auditory cortex.

In summary, the most frequent morphometric change is a decrease in GM in visual areas of the deaf pediatric population. Changes in the visual cortex in deaf adults and in auditory regions are less frequent and less consistent. Increases in GM have also been described in the cerebellum, especially at the right side, and in the hand region of the precentral gyrus.

### Changes in White Matter

Several studies reported decreases in WM in the temporal lobe in deaf adults and children. Seven studies reported WM decreases in the STG, either at the left side (Shibata, 2007; Olulade et al., 2014) or bilaterally (Emmorey et al., 2003; Kim et al., 2009; Smith et al., 2011; Feng et al., 2018; Kumar and Mishra, 2018). As for HG, three studies reported a left-lateralized decrease in WM (Shibata, 2007; Hribar et al., 2014; Olulade et al., 2014), whereas one study reported a bilateral decrease in WM (Emmorey et al., 2003). One other study reported a WM decrease within the anterior part of HG (Smith et al., 2011). In contrast, one study described increased WM volume around HG (Leporé et al., 2010a).

In deaf babies, WM decreases were described in the occipital lobe, either bilaterally (Feng et al., 2018) or in the left hemisphere only (Smith et al., 2011). A study in deaf adolescents reported a selective WM decrease in the right inferior occipital gyrus (Li J. et al., 2012).

Two studies described WM decreases in the left superior frontal gyrus (Kim et al., 2009) or in left middle frontal gyrus (Kim et al., 2009; Li J. et al., 2012) in deaf adolescents and adults. One study reported a WM increase in the left precentral gyrus and the right inferior frontal gyrus that was associated with the use of SL (Olulade et al., 2014). Deaf compared to hearing signers had a bilateral decrease in WM in the insula (Olulade et al., 2014). One other study reported a WM increase in the right insula in deaf and hearing signers (Allen et al., 2008).

Three studies reported a WM decrease within the cerebellum. In deaf babies, this WM reduction was either global (Feng et al., 2018), or limited to the region surrounding the crus II and lobule IX bilaterally (Smith et al., 2011). In deaf adults, this WM decrease covered the anterior lobe of the left cerebellum, and was just below statistical significance when corrected for multiple comparisons (Shibata, 2007).

A few studies also examined changes in long-range WM fiber tracts. One study in congenitally deaf adults reported a decrease of WM volume in the left superior longitudinal fasciculus (SLF) and left uncinate fasciculi (Meyer et al., 2007), but the effect disappeared after correction for multiple comparisons. Finally, two studies that focused on interhemispheric connections of the corpus callosum failed to show differences related to deafness (Kara et al., 2006; Leporé et al., 2010a).

In summary, the main modifications in WM in deaf individuals occurred in the temporal lobes, and more specifically around the STG which hosts the auditory cortex.

### Changes in Cortical Curvature

Only two studies investigated changes in cortical curvature in the auditory cortex. One study reported a steeper slope of the posterior Sylvian fissure (Meyer et al., 2014), whereas the second study failed to find an effect (Penhune et al., 2003).

### Results of the Activation Likelihood Estimation Meta-Analysis

**FIGURE 7:**
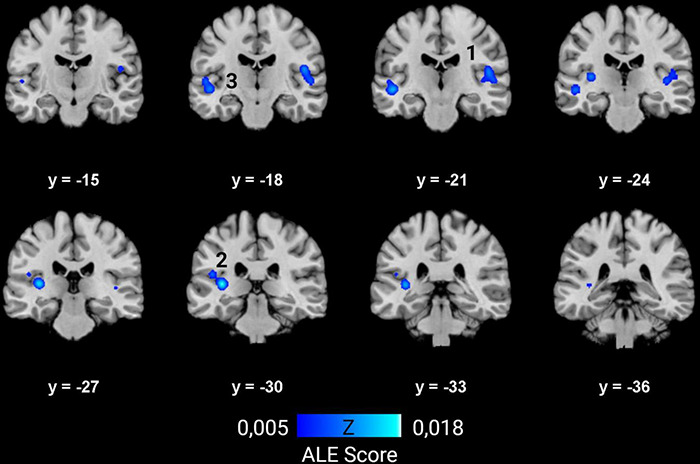
ALE meta-analysis of changes in GM and WM density in the deaf brain. Right hemisphere is shown on the right in the images. The numbers on top of the slices show the *y*-coordinates of the coronal slices in MNI space. Three clusters of decreased volume in deaf were found significant, and none of increased volume (*p* < 0.01, cluster-level family-wise error *p* < 0,05). They are situated in both STG and adjacent middle temporal gyrus and insula, and involve mainly WM. The numbers correspond to those indicated in Table 1.

Ten studies met the inclusion criteria for ALE, including the recent study of McCullough and Emmorey, for a total of 239 deaf subjects (68 children and 171 adults) compared to 289 normal hearing controls (82 children and 207 adults). All deaf participants except 30 presented with prelingual onset of deafness. The first dataset that we built gathers the coordinates of the peaks where deaf subjects showed greater volume than the normal hearing control subjects. They concern 30 foci from five studies (Leporé et al., 2010a; Hribar et al., 2014; Olulade et al., 2014; Kumar and Mishra, 2018; McCullough and Emmorey, 2021). The second dataset consists of 47 foci of lower volume in deaf, from eight studies (Kim et al., 2009; Smith et al., 2011; Li J. et al., 2012; Olulade et al., 2014; Feng et al., 2018; Kumar and Mishra, 2018; Qi et al., 2019; McCullough and Emmorey, 2021). Despite a relatively low number of studies, the meta-analysis highlights significant convergence in three clusters where deaf individuals have lower volume than their normal hearing controls. There is no cluster of increased volume in deaf subjects (Figure 7 and Table 1). Two clusters are located in the left hemisphere and one in the right. The clusters are mainly situated within the WM of both STG, including the HG, and the adjacent middle temporal gyrus and insula, and involve Brodmann Areas 13 (insula), 22 (posterior part of the STG, hosting Wernicke’s area in the left hemisphere) and 41 (anterior part of the HG, hosting the PAC). However, this coordinate-based meta-analysis has some potential biases. First, as mentioned above, only ten papers were included due to missing coordinates, whereas it is recommended to have at least 20 studies (Eickhoff et al., 2016). Second, the mix of pediatric and adult study cohorts, and to a lesser degree the mix of pre- and postlingual deafness, may increase heterogeneity of the results. Finally, the ALE methodology does not take into consideration the extent of the brain modification, leading to a lack of accuracy.

**TABLE 1:** Characteristics of the clusters from the ALE meta-analysis showing decreased volume in deaf subjects.

Clusters	Location	Tissue type	Related BA (relative weight)	Coordinates of the center (MNI)			Size (mm^3^)	Contributing studies
				*x-*	*y-*	*z-*		
1	R insula, STG, HG	52% WM	13 (26%), 41 (9%), 22 (7%)	49	−20	8	2152	Kim et al., 2009; Smith et al., 2011; Olulade et al., 2014; Kumar and Mishra, 2018
2	L STG, HG	75% WM	41 (16%), 13 (3%)	−37	−29	8	2016	Kim et al., 2009; Olulade et al., 2014; Feng et al., 2018; McCullough and Emmorey, 2021
3	L STG, MTG	71% WM	22 (21%), 13 (4%)	−50	−20	−4,5	1552	Smith et al., 2011; Olulade et al., 2014; Kumar and Mishra, 2018

*MNI coordinates, P < 0.05 cluster-level FWE. BA, Brodmann area; R, right; L, left; STG, superior temporal gyrus; HG, Heschl’s gyrus; MTG, middle temporal gyrus; WM, white matter.*

## Discussion

### Structural Brain Changes in Human Deafness

#### Overview of the Results

The three most consistent findings in deaf subjects derived from our review are (1) a volumetric decrease in WM in auditory cortex; (2) a volumetric decrease in GM and WM in visual cortex, particularly in babies and children; and (3) a GM increase in the right cerebellum. Cortical thickness and curvature have been studied sparsely in comparison with GM and WM volume, making it difficult to draw firm conclusions with respect to these measures.

Unlike a recent meta-analysis (Manno et al., 2021), we have chosen to focus exclusively on severe and profound congenital and acquired hearing loss, excluding other degrees of hearing impairment. A large part of the published studies focused on plastic changes in early deaf subjects, i.e., congenital and acquired prelingual deafness, for whom SL is typically the primary language. On the contrary, most studies on postlingual deafness include elderly people with only mild to moderate age-related hearing loss (Manno et al., 2021).

#### Impact of the Language

As most prelingually deaf adults use SL as their only language, the comparison with normal hearing subjects can introduce some bias due to the difficulty in distinguishing the effects of deafness from those of SL (Cardin et al., 2013). Only a few studies from our literature review dealt with this issue by including an extra control group of normal-hearing individuals who were fluent in SL (Fine et al., 2005; Allen et al., 2008, 2013; Olulade et al., 2014; McCullough and Emmorey, 2021). These studies revealed that SL use *per se* induces brain modifications, especially in the hand motor region, and in regions involved in visual processing of faces and hands during SL comprehension (see in subsequent sections for more details). Furthermore, the age of SL acquisition also plays a central role in brain morphology, corroborating the idea of a critical period of language acquisition, such as for the development of the auditory brain. For example, the lack of early SL access in case of deafness is responsible for a GM decrease within the occipital lobe (Pénicaud et al., 2013) and for microstructural alterations of the arcuate fasciculus (AF), which is part of the AF-SLF complex (Cheng et al., 2019). The AF-SLF complex, whose anatomical classification is still debated, is involved in language processing, as well as the inferior fronto-occipital fasciculus (IFOF) and the uncinate fasciculus (Figure 2). More specifically, the IFOF and uncinate fasciculus belong to the ventral language pathway which plays a critical role in semantic language processing, goal-oriented behavior, and visual task switching (Conner et al., 2018). On the other hand, the AF-SLF complex is part of the dorsal language pathway that is involved in syntax and speech repetition (Dick et al., 2014). This dorsal pathway also participates in the visuospatial attention network, and could more largely be involved in attentional control across multiple sensory modalities (Chechlacz et al., 2013). The alteration of the AF-SLF complex in late SL learners could explain the abnormal development of neuro-linguistic structures in the brain, affecting especially grammar and second language acquisition (Skotara et al., 2012). Indeed, a first language acquired in infancy facilitates the learning of a second language, independently of the modality of the first or second language (oral or signed) (Mayberry et al., 2002). This highlights the great importance of providing language tools to deaf babies to enable the development of related WM tracts and facilitating potential further adaptation to another language modality, for example after CI rehabilitation. Moreover, the lack of language access is responsible for impaired cognitive and socioemotional development (Cheng and Mayberry, 2019), causing cognitive delays, mental health difficulties and lower quality of life (Hall, 2017).

In summary, the modality of the language and its age of acquisition have an important impact on brain structure and function. Indeed, exposure to any language at a young age allows the normal development of the “language brain,” especially its white matter bundles. This highlights the importance of hearing screening to be able to rapidly offer language, either oral after cochlear implantation or visual. Moreover, further studies are needed to explore the role of oral and visual language, e.g., SL and speech reading, on brain anatomy and CI rehabilitation.

#### Structural Changes in Auditory Brain Areas

The most conspicuous change was a decrease of WM density within the STG, which hosts the auditory cortex and part of Wernicke’s speech area, in both children and adults. This finding is highlighted by the ALE meta-analysis that showed three clusters of decreased density in the deaf brain, mainly in WM, around the STG and insula. The results for GM modifications were more ambiguous. The temporal lobe in prelingually deaf adults exhibited an increase of GM density in the STG, especially in the right hemisphere, and bilaterally in the inferior temporal gyrus. In children, the results were inconsistent, some reporting increases and others decreases in GM in STG. A recent study conducted in a large cohort of 94 postlingually deaf adults, not included in the ALE analysis because of lack of MNI coordinates, found a global GM decrease in the superior, middle and inferior temporal cortices (Sun et al., 2021). This study further showed an interaction between GM changes and duration of deafness: the decrease in the middle temporal cortices was found exclusively in participants who had been deaf for more than 10 years, whereas the decrease in the superior temporal cortices was limited to participants who had been deaf for less than 10 years. In addition to demonstrating that neuroplastic changes in postlingually deaf subjects evolve with duration of deafness, authors found correlation between specific GM modifications and speech comprehension after CI rehabilitation.

The WM decrease combined with the GM increase in prelingually deaf subjects suggests that early lack of auditory stimulation interferes with normal cortical GM and WM maturation in PAC, resulting in less myelination, fewer fibers projecting to and from auditory cortices, increased and inadequate axonal pruning, and incomplete neuronal migration (Emmorey et al., 2003; Penhune et al., 2003; Smith et al., 2011; Kumar and Mishra, 2018). Diffusion Tensor Imaging (DTI) studies confirmed the alteration of WM around the STG in prelingually [see Hribar et al. (2020) for reviews] and postlingually deaf subjects (Li Y. et al., 2012). More generally, the different relays of the auditory pathways of early deaf demonstrated a decrease in fractional anisotropy (Miao et al., 2013; Hribar et al., 2014; Huang et al., 2015; Wu et al., 2016; Karns et al., 2017) [see Tarabichi et al. (2018) for a review], which is deleterious for CI rehabilitation (Wu et al., 2009; Chang et al., 2012; Huang et al., 2015). In congenitally deaf teenagers and adults, decreases in FA were also found in the IFOF, SLF and uncinate fasciculus (Kim et al., 2009; Miao et al., 2013; Hribar et al., 2014) [see Simon et al. (2020) for a review], although to a lesser extent. No such changes have been demonstrated in pediatric or postlingually deaf individuals. Interestingly, a recent study found that early language acquisition, whether oral or signed, enabled the normal development of the different WM bundles involved in language, whereas microstructural WM alteration were found within the AF-SLF complex in congenitally deaf adults with late SL acquisition (Cheng et al., 2019). Taken together, the modifications of these WM bundles could be due to oral language deprivation, to SL, or even to early language deprivation in case of delayed diagnosis of deafness. Furthermore, the alteration of the AF-SLF complex may be linked to deficits in executive functions, for instance memory and attention, demonstrated in deaf children and adults (Kronenberger et al., 2014; Kramer et al., 2018).

#### Structural Changes in the Frontal Lobe

Structural changes have been reported in the inferior frontal gyrus, involved in linguistic and cognitive functions such as text reading, speech production and working memory (Friederici and Gierhan, 2013), and also in other parts of the frontal lobe dealing with cognitive functions or hand movements. However, SL use seems to play a central role in the brain modifications of the frontal lobe, and further research with respect to this issue is needed. The GM increase in the inferior frontal gyri could be due to increased demand for deciphering oral language through print and lip-reading, and to increased reliance on visual working memory processes in the prelingually deaf (Allen et al., 2013). On the other hand, the volume and microstructure of the WM was altered in the inferior, middle and superior frontal gyri, as confirmed by some DTI studies (Chang et al., 2012; Zheng et al., 2017). This is in line with the alteration of SLF and IFOF, as discussed above. On the contrary, SL rather induces WM volume increase in the left precentral and right inferior frontal gyrus (Olulade et al., 2014).

Surprisingly, a recent meta-regression study (Manno et al., 2021) concludes that deaf subjects present a GM decrease in the frontal lobe, which is in contradiction with our results and those from other reviews (Hribar et al., 2020; Simon et al., 2020).

#### Structural Changes in the Visual Areas

The occipital cortex is the brain area with the second most structural modifications related to deafness. Especially in babies and children, decreases in GM volume and thickness, and to a lesser extent in WM volume, were observed in primary, secondary and high-level visual areas. The results in deaf adults are less consistent, perhaps due to differences in the age of acquisition of SL. Indeed, as discussed above, prelingually deaf adults with late SL acquisition present a GM decrease in primary, secondary and higher order visual association areas, whereas those with early SL acquisition have increased GM within these areas (Pénicaud et al., 2013). The important role of SL is corroborated by a recent study showing that lifelong signing experience is associated with a reduction in cortical thickness in the right occipital lobe and with an expansion in the surface area of the left occipital lobes (McCullough and Emmorey, 2021). This study also reported an expansion of the surface of the left anterior temporal lobe in SL users. All these changes were attributed to the high demands of processing and integration of visual information from the face and hands during SL comprehension. The well-documented enhanced peripheral vision of deaf subjects could also play a role in the modifications in the occipital lobe (Neville and Lawson, 1987b). However, enhanced peripheral vision may have a negative effect on central visual attention and may be responsible for increased distractibility in a central visual task with peripheral distractors (Quittner et al., 2004; Bavelier et al., 2006).

The results for the fusiform area are inconsistent, some authors reporting a GM density increase or decrease, others a cortical thickness decrease (Li J. et al., 2012; Olulade et al., 2014; Kumar and Mishra, 2018; Qi et al., 2019), and still others reporting no changes. The fusiform gyrus is a high-level visual area involved in face perception, object recognition and reading (Çukur et al., 2013; Weiner and Zilles, 2016). A DTI study showed that a decrease in GM or cortical thickness of the fusiform gyrus is associated with alterations in the IFOF (see above) and the inferior longitudinal fasciculus (Qi et al., 2019). Indeed, the inferior longitudinal fasciculus is involved in object recognition and face perception (Wang et al., 2020). The fact that the fusiform gyrus shows more GM decreases in the left compared to the right hemisphere could be explained by the stronger reliance on phonology and the increased engagement of the right hemisphere in visual word processing in deaf readers (Emmorey and Lee, 2021). On the other hand, Kumar and co-workers showed a GM increase in the fusiform and inferior temporal gyri which they attributed to a stronger reliance on ventral and higher visual processing in deaf individuals (Kumar and Mishra, 2018). Finally, the GM increase in the right lateral geniculate nucleus could be due to the stronger reliance on visual information in deaf subjects, which will also be processed in the right auditory cortex (Finney et al., 2001; Almeida et al., 2015; Amaral et al., 2016).

#### Structural Changes in the Cerebellum

Prelingual deafness induces anatomical changes within the cerebellum, especially a GM increase mostly at the right side in crus I and II, lobules IV–V and IX–X (Leporé et al., 2010a; Li et al., 2013; Hribar et al., 2014; Kumar and Mishra, 2018), and a GM decrease in the left crus II and lobule VIII (Olulade et al., 2014). The WM volume was decreased in deaf babies, especially around the crus II and the cerebellar tonsils or lobules IX bilaterally (Smith et al., 2011; Feng et al., 2018).

The cerebellum is best known for its role in motor control and planning. The sensorimotor cerebellum is located in anterior lobules III to V, and in lobules VI and VIII; it is functionally connected with the contralateral cerebral sensorimotor cortices (Stoodley and Schmahmann, 2009; Buckner et al., 2011). Since the hand is represented in lobule V (Grodd et al., 2001), the GM increase in this area could be explained by increased demands in fine motor coordination of hand movements during SL.

The cerebellum also controls balance and posture by integrating vestibular and sensorimotor inputs. In particular, vermal lobule IX, lobule X (flocculus and nodulus), and vermal lobules I and II (lingula) receive afferents from the vestibule (Stoodley and Schmahmann, 2009). Therefore, vestibular dysfunction could also induce neuroplastic changes in the cerebellum of deaf subjects. It is important to notice that more than 50% of congenitally deaf subject present some vestibular dysfunction (Kaga et al., 2008). This is not surprising since the cochlear and vestibular organs share anatomical, histological and physiological similarities (Cushing et al., 2013).

Finally, the cerebellum is also involved in many cognitive processes such as verbal working memory, phonological storage, sound and speech recognition, attention, spatial tasks, visual perception of motion, speed and direction, and affective regulation (Ivry and Diener, 1991; Middleton and Strick, 1994; Glickstein, 2007; Sens and De Almeida, 2007; Grimaldi and Manto, 2012; Mariën et al., 2014; McLachlan and Wilson, 2017). Some of these higher-level tasks, such as language and working memory, activate the right posterolateral lobe, which has increased GM in prelingually deaf (Stoodley and Schmahmann, 2009; Li et al., 2013). In addition to its role in spoken language, the cerebellum is even more strongly involved in the production and comprehension of SL (Kassubek et al., 2004; Sakai et al., 2005). Taken together, the cause of the changes in cerebellar GM and WM could be multifactorial, and be related to SL production and comprehension, modified cerebellar auditory and vestibular processing, deficits in memory and attention, stronger reliance on higher cognitive tasks such as deciphering spoken language through lip-reading, the use of visual cues for emotion recognition (Baumann and Mattingley, 2012) or visual working memory.

#### Structural Changes in the Insula

The reported volumetric changes in the insula vary considerably and range from a GM increase in the posterior left insula (Allen et al., 2008) to an overall bilateral GM decrease (Olulade et al., 2014). A recent study reported a GM decrease in the right insula in postlingual deafness (Sun et al., 2021), a finding which was confirmed in a meta-analysis and meta-regression study (Manno et al., 2021). Conflicting results were also reported for the insular WM, going from a bilateral decrease in deaf compared to normal hearing signers (Olulade et al., 2014) to a WM increase in the right insula in SL users (comparison between deaf and normal hearing signers, and normal hearing non-signers) (Allen et al., 2008). This suggests that deafness and SL exert different effects on the insula.

It has been hypothesized that the strong reliance of deaf individuals on lip-reading and articulatory-based representations of speech could impact the structure and the function of the insula (Allen et al., 2008). For instance, fMRI studies highlight an enhanced connectivity between the insula and auditory cortex or superior parietal gyrus, which could support the increased reliance on cross-modal integration in deaf subjects (Ding et al., 2016; Li et al., 2016). Moreover, deaf subjects show enhanced recruitment of the insula and thalamus during verbal memory tasks (Bavelier et al., 2008).

#### Negative Results

One third of the studies did not find any significant modifications in brain anatomy. Several hypotheses can be put forward to explain these negative findings. First, more than half of the included studies have a sample size of less than 20 subjects per group, resulting in low statistical power (Smith et al., 2011; Kim et al., 2014; Li et al., 2015). This is particularly the case for VBM whole brain analysis that requires larger numbers of subjects in order to reach statistical significance (Shibata, 2007). In line with this, studies that have shown GM changes in the temporal lobe were among those with the most participants. Second, the use of an univariate approach (e.g., measure of volume) instead of a multivariate one (e.g., measure of cortical thickness, surface, density, and curvature) reduces the chances to detect modifications, as they focus on one specific characteristic of brain tissue only (Kim et al., 2014; Ratnanather, 2020). Third, morphometric analyses like VBM and tensor-based morphometry (TBM) allow only evaluation of macrostructural alterations, whereas diffusion imaging enables to detect microstructural changes in WM. For example, in case of early deafness, only four out of 17 VBM studies found WM decrease in the STG, compared to 80% of studies using DTI (Simon et al., 2020).

### Comparison With Animal Model of Deafness

Brain modifications in deafness have been more consistently reported in animal studies than in human studies. Some similar changes have been described in both animal and human studies. For instance, at the level of the cochlear nuclei, a decrease in the size of the cell bodies has been reported in histological studies in both animals and humans (Hultcrantz et al., 1991; Niparko and Finger, 1997; Hardie and Shepherd, 1999; Chao et al., 2002). MRI studies in deaf humans reported a decrease of WM density in the STG which is in line with a volumetric reduction of the infragranular cell layers of the auditory cortex in deaf animals, where the efferent fibers derive from. At the level of the visual cortex, GM volumetric decreases in V1 were consistently reported in deaf animals and in deaf human babies but not in prelingually deaf adults, possibly due to the use of sign-language. On the other hand, in contrast to animal studies, studies in human deaf individuals failed to find a global atrophy of the PAC. This could be due to the use of non-invasive imaging techniques in human studies which are less specific and suffer from a poorer spatial resolution (Moerel et al., 2014) compared to cytoarchitectonic methods used in animal studies. Although recent studies using ultrahigh-field (7 Tesla) multi-modal brain imaging techniques and novel methods for intersubject alignment have led to better probabilistic atlases of the human auditory cortex (Gulban et al., 2020), the exact delimitation of the boundaries of the human auditory cortex is still unresolved (Hackett et al., 2001; da Costa et al., 2011; Moerel et al., 2014). Another potential explanation for the lack of cortical atrophy in the STG in human studies is the use of visual-based SL instead of speech, which allows to maintain the language function of some specific areas (Neville et al., 1998; Anderson et al., 2017; Cardin et al., 2020).

### Comparison With Visual Deprivation

Brain structural and functional changes following loss of vision have been studied in far greater detail than those following auditory deprivation [see Kupers and Ptito (2014) for a review]. These studies highlighted that the deprived visual cortex becomes sensitive to other sensory modalities, including, tactile, auditory and olfactory inputs, leading to superior skills in some perceptual tasks (Bavelier and Neville, 2002; Burton et al., 2003, 2004; Chebat et al., 2007b; Renier et al., 2014; Araneda et al., 2016). These functional changes are accompanied with vast structural changes, including GM volume reductions in the different relays of the visual pathways, such as the superior colliculus, lateral geniculate nucleus, posterior pulvinar, primary and secondary visual cortices (Noppeney et al., 2005; Shimony et al., 2006; Pan et al., 2007; Ptito et al., 2008; Jiang et al., 2015; Cecchetti et al., 2016; Touj et al., 2021). The reductions in GM volume in visual areas could be the result of deprivation-related disuse (Noppeney et al., 2005; Voss et al., 2014). Reductions in WM of the visual tracts were reported in the optic nerve, optic chiasm and optic tract (Noppeney et al., 2005; Shimony et al., 2006; Pan et al., 2007; Ptito et al., 2008, 2021; Bridge et al., 2009; Leporé et al., 2010b; Tomaiuolo et al., 2014), the posterior part of the corpus callosum (Tomaiuolo et al., 2014) and the anterior commissure (Cavaliere et al., 2020). Several studies also reported increased cortical thickness of the primary visual cortex in early-blind subjects (Bridge et al., 2009; Jiang et al., 2009; Voss and Zatorre, 2012; Qin et al., 2013), which might be explained by a reduction of synaptic pruning due to lack of visual experience (Huttenlocher, 1984; Park et al., 2009; Kupers and Ptito, 2014; Anurova et al., 2015).

Some GM volume increases were also demonstrated outside the visual areas, for example in hippocampus (Chebat et al., 2007a; Fortin et al., 2008), sensorimotor areas (Jiang et al., 2015), the olfactory bulb, olfactory nucleus and piriform cortex (Rombaux et al., 2010; Touj et al., 2021), amygdala (Touj et al., 2021), and right inferior parietal cortex (Bauer et al., 2017). On the other hand, no studies showed structural changes in the auditory cortex of blind individuals (Noppeney et al., 2005; Pan et al., 2007; Ptito et al., 2008), except one which found a decrease in cortical thickness (Park et al., 2009).

Several hypotheses can be put forward to explain why structural changes are less prominent in case of deafness. A first explanation is that the visual system takes a much more prominent role in the human brain compared to audition. An estimated 30–40% of the cortical mantle is devoted to processing visual information, compared to only 8% for audition (Wandell et al., 2007). A second explanation is that studies investigating congenital blindness nearly all exclude individuals with residual vision, including light perception. This is in sharp contrast with studies on (congenital) deafness which uses a less stringent criterion, defining deafness in case of severe or profound hearing loss with auditory thresholds greater than 70 dB HL. In other words, these individuals still have remaining auditory capacities, although very limited. In this sense, deafness is more reminiscent to the categories of “low vision” or “legally blind,” i.e., individuals with a visual acuity of 20/200 or less in the best eye, while wearing corrective glasses or contacts. Another hypothesis is that the potential structural modifications in the STG due to auditory deprivation are limited because this area continues to process language in the visual modality (Neville et al., 1998; Anderson et al., 2017; Cardin et al., 2020). Finally, there is ample anatomical and physiological evidence that auditory processing is strongly modulated by visual and somatosensory input (Bizley et al., 2007; Kayser et al., 2008; Smiley and Falchier, 2009; Banks et al., 2011; Ro et al., 2013; Meredith and Allman, 2015). The integration of auditory with visual and somatosensory input takes place at each level of the ascending auditory pathway, including the cochlear nucleus, inferior colliculus, medial geniculate body and the auditory cortex [for review: Wu et al. (2015)]. Therefore, the important multisensory input to auditory brain structures may explain why auditory brain areas are less affected by auditory deprivation.

### Limitations and Perspectives

The included papers suffer from some limitations which should be considered in future investigations. First, the included papers attributed the reported brain modifications to deafness, omitting the possibility that other factors which can interact with cerebral morphology, such as etiology of the deafness (e.g., genetic, infectious, and due to medication), SL and hearing aids use, vestibular dysfunction, neurocognitive skills, etc. For example, the use of hearing aids may by itself induce functional and structural brain changes in auditory and language-related areas, the associative regions and the cerebellum (Li et al., 2013; Pereira-Jorge et al., 2018). Second, the exclusive reliance on non-oral communication in the prelingually deaf makes it difficult to match the deaf and control populations in terms of psychosocial and socio-economic variables. Indeed, most tools used for intellectual evaluation are based on oral communication, and moreover deaf subjects have less opportunities of professional training and inclusion. Third, some of the sample sizes are low which reduces the statistical power. Finally, whereas various metrics can be taken in morphometric analyses, such as volume, surface, cortical thickness, and curvature, most of the papers focused on an univariate approach. Although these metrics are interrelated (f.i., cortical volume is the product of cortical surface and thickness), they measure different aspects of the same cerebral region; the measure of cortical thickness and surface independently provides the best appreciation of the brain modifications (Ratnanather, 2020).

This review has also its proper limitations. First, the existing literature meeting our inclusion criteria concerned almost exclusively studies in prelingually deaf subjects, at the detriment of the postlingually deaf population. The study of prelingually deaf population presents some advantages linked to the homogeneity of this population with respect to the age of onset of deafness, the severity of the hearing loss, and the use of SL as primary language in absence of effective hearing aids. Mild to moderate hearing loss in the aging population has been more frequently studied, but studies of severe to profound hearing loss are lacking. This is more surprising since this population is larger than the prelingually deaf population (Ratnanather, 2020), and will continue to grow due to an aging population. In this specific group, the frequent association with dementia or tinnitus should also not be forgotten. A second limitation of our review is the choice to concentrate on macrostructural brain modifications using 3D-T1-weighted MRI. Indeed, diffusion imaging provides more qualitative and quantitative information about WM microstructure. A third limitation relates to the absence of well-established anatomical limits of functional regions; these can differ from one brain atlas to another. Inter-individual variability in brain anatomy can also complicate the comparisons. For instance, in case of duplicated HG, authors considered that the PAC was situated on the most anterior gyri, while it has since been demonstrated that it spans both divisions of HG (da Costa et al., 2011). When hand-drawn ROIs are used, the delineation of anatomical areas is also susceptible to be biased by assumptions of what form the ROI should have (Shibata, 2007; Li J. et al., 2012). The use of stereotactic coordinates is certainly the most accurate method, and these one should be shared in the papers.

This review demonstrates that severe to profound prelingually deafness is responsible of structural brain modifications mainly but not limited to the temporal lobe. Our results are also of clear clinical interest, since brain plastic changes can facilitate, or complicate, CI auditory rehabilitation [for example Feng et al. (2018) or Sun et al. (2021)]. In contrast, regions unaffected by deafness, such as the fronto-parietal network and dorsal lateral or medial prefrontal cortex, showed a high predictive power. Another example where brain plasticity can predict CI outcome concerns lip-reading abilities. The use of lipreading, instead of written language, allows to maintain phonological representations and left hemispheric language specialization, which further increase speech comprehension (Lazard and Giraud, 2017). This teaches us that lip-reading must be encouraged in the deaf population. It would be very interesting to correlate this finding with structural or functional brain imaging. Finally, diffusion and functional imaging can also give some predictive factors of the speech outcomes with a CI. For example, preoperative inferior colliculus FA values correlate positively with postoperative auditory performance in deaf children (Wang et al., 2019). These examples highlight the potential contribution of pre-operative MRI to CI professionals as a clinical outcome prediction tool. It can also guide clinicians to adapt their strategy of care, for example by training lip-reading to avoid maladaptive plasticity.

## Conclusion

Severe to profound deafness induces modifications of both brain GM and WM characteristics. The major modifications are a WM decrease around the auditory cortex, the occipital lobe and the cerebellum, a GM decrease in the occipital lobe of the deaf pediatric population, and a GM increase of the right cerebellum. Different measures (volume, surface, curvature, and cortical thickness) and methods (VBM, TBM, and DTI) should be combined to create a more comprehensive view of the brain modifications. Deaf subjects must be better categorized to dissociate brain changes due to deafness from those of sign language use, age of SL acquisition, lip-reading abilities, etiology of deafness, use of hearing aids, and vestibular dysfunction. More attention needs to be paid to structural changes in postlingual deafness which affects the vast majority of the deaf and aging population in western countries.

## Data Availability Statement

The original contributions presented in the study are included in the article/Supplementary Material, further inquiries can be directed to the corresponding author.

## Author Contributions

AG did the literature review and wrote the first draft of the manuscript. AG, ND, and RK wrote the final version of the manuscript. All authors contributed to conception and design of the study, manuscript revision, read, and approved the submitted version.

## Conflict of Interest

The authors declare that the research was conducted in the absence of any commercial or financial relationships that could be construed as a potential conflict of interest.

## Publisher’s Note

All claims expressed in this article are solely those of the authors and do not necessarily represent those of their affiliated organizations, or those of the publisher, the editors and the reviewers. Any product that may be evaluated in this article, or claim that may be made by its manufacturer, is not guaranteed or endorsed by the publisher.

## Abbreviations

CI: cochlear implant
DTI: diffusion tensor imaging
HG: Heschl’s gyrus
IFOF: inferior-fronto-occipital fasciculus
PAC: primary auditory cortex
ROI: region of interest
SLF: superior longitudinal fasciculus
STG: superior temporal gyrus.
