# Corrigendum: Brain morphological modifications in congenital and acquired auditory deprivation: a systematic review and coordinate-based meta-analysis

**DOI:** 10.3389/fnins.2024.1354571

**Published:** 2024-01-22

**Authors:** Anaïs Grégoire, Naïma Deggouj, Laurence Dricot, Monique Decat, Ron Kupers

**Affiliations:** ^1^Department of ENT, Cliniques Universitaires Saint-Luc, Brussels, Belgium; ^2^Institute of NeuroScience (IoNS), UCLouvain, Brussels, Belgium; ^3^Department of Neuroscience, Panum Institute, University of Copenhagen, Copenhagen, Denmark; ^4^Ecole d'Optométrie, Université de Montréal, Montréal, QC, Canada

**Keywords:** deafness/hearing loss, sign language (SL), neuroplasticity, brain morphology, cochlear implant, MRI, systematic review and meta-analysis

In the published article, there was an error. In the abstract, “grey matter” has been written instead of “white matter”.

A correction has been made to the abstract. The sentence previously stated:

“The most consistent finding is a volumetric decrease in gray matter around bilateral auditory cortex”.

The corrected sentence appears below:

“The most consistent finding is a volumetric decrease in white matter around bilateral auditory cortex”.

The authors apologize for this error and state that this does not change the scientific conclusions of the article in any way. The original article has been updated.

