# Supplementary material for: Brain Morphological Modifications in Congenital and Acquired Auditory Deprivation: A Systematic Review and Coordinate-Based Meta-Analysis
Source: Front Neurosci. 2022 Mar 28;16:850245. doi: 10.3389/fnins.2022.850245 (PMC8995770; doi:10.3389/fnins.2022.850245)
Supplement: Supplementary file 1 [file Data_Sheet_1.pdf]

Table 1. Demographic characteristics and main findings in the deaf population compared to normal hearing subjects.

References	Area of investigation	Method	Participants characteristics				Deafness characteristics				Statistical differences
			N°	Mean age ± SD (Range)	Sex (F/M)	Handedness	Severity (number)	Onset, language	Hearing aids	Etiology	
Allen J, 2008 *	insula	manual volumetry	25 D<br>16 HS<br>25 H	23,8 ± 4,1 (19-38)<br>24,3 ± 4,4 (19-38)<br>28,5 ± 4,5 (22-39)	38/28	RH<br>RH<br>RH	> 90 (21), > 75 dB (3), > 55 dB (1)	preL, SL	yes for some, after 2 years old	NS	GM: ↑ L post insula in D vs H<br>WM: ↑ R insula in D and HS vs H
Allen J, 2013 *	CS, pars triangularis and opercularis, motor region	manual volumetry	25 D<br>16 HS<br>25 H	23,8 ± 4,1 (19-38)<br>24,3 ± 4,4 (19-38)<br>28,5 ± 4,5 (22-39)	38/28	RH<br>RH<br>RH	> 90 (21), > 75 dB (3), > 55 dB (1)	preL, SL	yes for some, after 2 years old	NS	GM: ↑ total CS in D vs H, ↑ L CS in D vs H, R CS larger than the L in all groups; ↑ bilat pars triangularis in D vs H and HS
Amaral L, 2016 *	Thal nuclei, IC, SC	manual volumetry	15 D<br>16 H	20,4 (17-22)<br>20,1 (18-22)	26/5	RH<br>RH	> 90 dB	preL, SL	no	NS	GM: ↑ R vs L thal, lat gen nucl and IC
Emmorey K, 2003 *	temp lobe, STG, HG, PT	manual volumetry	25 D<br>25 H	23,8 ± 4,1 (19-38)<br>28,5 ± 4,5 (22-39)	28/22	RH<br>RH	> 90 (21), > 75 dB (3), > 55 dB (1)	preL, SL	yes for some, after 2 years old	NS	GM: ↑ R STG and PT<br>WM: ↓ bilat HG and STG
Feng G, 2018	WB	VBM and MVPS (GM, WM)	37 D<br>40 H	17,9 months ± 7,81 (8-38)<br>18 months ± 10,2 (8-38)	32/45	/<br>/	> 70 dB (27), > 50 dB (10)	preL, /	yes	NS	GM: ↓ bilat STG, HG, parahippocampal gyrus, L medial FG, L precuneus, L lingual gyrus, R supramarginal gyrus, R middle Cing, R SOG<br>WM: ↓ bilat STG, front ant region, occ lobe, cerebellum
Fine I, 2005	early visual cortex (V1-V4), motion area (MT), defined by fMRI	VBM (GM, WM)	6 D<br>6 HS<br>6 H	27 ± 5,7<br>23,5 ± 6,3<br>26,8 ± 2,6	9/9	RH<br>RH<br>RH	> 80 dB	preL, SL	NS	NS	GM: no diff<br>WM: no diff<br>WM: no diff
Hribar M, 2014	HG, WB	CT, VBM, surface-based analysis (GM)	14 D<br>14 H	35,4 ± 6 (23-50)<br>30,5 ± 5,2 (23-50)	16/12	RH<br>RH	> 90 dB	preL, SL	no	NS	GM: ↑ cerebellum<br>WM: ↓ L HG
Kara A, 2006	CC	manual volumetry	18 D<br>18 H	41,2 ± 7,5<br>36,4 ± 7,1	0/36	RH<br>RH	> 90 dB	preL, SL	NS	NS	GM: /<br>WM: no diff
Kim D, 2009	WB	VBM (WM)	13 D<br>29 H	29,3 ± 6,8<br>26,5 ± 4,5	19/23	RH<br>RH	> 90 dB	preL, NS	"not for brain developmental period"	fever, meningitidis, auditory nerve atrophy	GM: /<br>WM: ↓ bilat STG and temp sub-gyral areas, L pariet, L SFG and L medial FG
Kim E, 2014	primary auditory cortex	VBM (GM)	8 D<br>11 D<br>11 H	50,4 ± 6,1<br>50,9 ± 12,2<br>49,5 ± 8,9	15/15	RH<br>RH<br>RH	> 70 dB<br>>70 dB	preL, SL<br>postL, oral	no<br>yes	NS	GM: no diff
Kumar U, 2018	WB	VBM (GM, WM), source-based [illegible]	50 D<br>50 H	19,5<br>19,5	matched but NS	matched but NS	> 90 dB	preL, oral	no	NS	GM: ↑ bilat MFG, bilat ITG, bilat fus, R cerebellum, L suppl motor area, R inf pariet region, ↑ CT bilat STG<br>WM: ↓ bilat STG
Leporé N, 2010	CC, lobes, WB	tensor-based morphometry (GM, WM)	14 D<br>16 H	29,5 (21-52)<br>24	15/15	RH<br>RH	> 90 dB	preL, SL	NS	NS but no CMV or meningitidis	GM: ↑ STG, R cerebellum, R MFG, L IFG, bilat preC<br>WM: ↑ bilat HG and intraparietal areas
Li J, 2012 °	WB	VBM (GM, WM), CT	16 D<br>16 H	14,56 ± 2,10 (10-18)<br>14,75 ± 2,38 (10-18)	16/16	RH<br>RH	> 90 dB	preL, SL	yes	drug toxicity (9), genetics (7)	GM: ↓ CT WB, L preC, R postC, L SOG and L fus gyrus<br>WM: ↓ L middle front gyrus and R inf occ gyrus
Li W, 2013 °	WB	VBM (GM, WM), CT	16 D<br>16 H	14,56 ± 2,10 (10-18)<br>14,75 ± 2,38 (10-18)	16/16	RH<br>RH	> 90 dB	preL, SL	yes	drug toxicity (9), genetics (7)	GM: ↑ Rw as of density in cerebellum, ↑ Lw as of CT in post Cing, ↑ Lw as of CT in gyri rectus (↑ L and ↓ R), ↑ Rw as of CT in precuneus, ↑ Rw as of CT of MFG (↓ L), ↑ Rw as of CT SFG (↓ L and ↑ R), ↑ Rw as of CT MOG<br>WM: no diff
Li W, 2015 °	14 ROI within temp, front, parietal, occ gyri	manual volumetry	16 D<br>16 H	14,56 ± 2,10 (10-18)<br>14,75 ± 2,38 (10-18)	16/16	RH<br>RH	> 90 dB	preL, SL	yes	drug toxicity (9), genetics (7)	GM: no diff
Meyer M, 2007	post Sylvian fissure, WB	manual curvature,	6 D	23,5 (19-43)	4/8	RH	NS	preL, SL	NS	NS	*sleeper slope of the post Sylvian fissure*

		VBM (GM, WM)	6 H	26,67 (22-45)		RH					WM: no diff
Olulade O, 2014 *	WB	VBM (GM, WM)	15 D<br>15 D<br>15 HS<br>15 H	23,4 ± 3,3 (18,4-31,8)<br>28,2 ± 3,8 (22,8 - 34,6)<br>26,7 ± 6,9 (18,4 - 39,5)<br>25,9 ± 6,0 (18,6 - 41,8)	32/28	RH<br>RH<br>RH<br>RH	> 75 dB<br>> 75 dB	preL, SL<br>preL, oral	NS	NS	GM: *D SL user vs HS* : ↑ R SFG, ↓ bilat STG, HG and insula, ↓R claustrum, R fus, R inf pariet and L MTG, *D vs H* : ↑ bilat MTG, SFG and R MFG, ↓ L ant fus, lingual gyri, cerebellum, and R post Cing, *SL vs English* : ↑ R medial FG, MFG and IFG and precuneus, L MFG and Cing<br>WM: *D SL user vs HS* : ↓ bilat STG, HG, MTG, ITG, bilat fus and insula, ↓ L parahippocampal gyri; *D vs H* : ↓ L STG and HG; *SL vs English* : ↑ L preC and R IFG
Penhune V, 2003	HG, PT, WB	manual volumetry, VBM (GM, WM)	15 D<br>10 H	29<br>32	10/12	RH<br>RH	> 90 dB	preL, SL	NS	NS	GM: no diff<br>WM: no diff
Pénicaud S, 2013	WB	VBM (GM, WM)	15 D<br>43 H	39,2 ± 12,3 (25-61)<br>37,3 ± 11,5 (25-62)	33/33	RH<br>RH	> 90 dB (18), > 80 dB (5)	preL, SL	NS	NS	GM: ↓ L V1, V2 and V3a/V7 in late SL acquisition, ↑ L V1, V2, V3a/V7 in SL acquisition during infancy<br>WM: ↑ LV3a/V7 in late SL acquisition
Qi R, 2019	WB	VBM (GM)	35 D<br>23 H	39,72 ± 1,81<br>39,83 ± 1,96	23/35	NS<br>NS	> 90 dB (33), > 40 dB (2)	preL, NS	NS	Congenital (31), drug toxicity (2), infectious disease (2)	GM: ↓ R fus and R MOG<br>WM: /
Shi B, 2016	WB	VBM (GM, WM)	15 D<br>12 H	9,36 ± 3,14 (5-13)<br>8,41 ± 2,67 (5-14)	9/14	NS<br>NS	> 90 dB	preL, SL	NS	NS	GM no diff<br>WM: /
Shibata D, 2007	WB	VBM (GM, WM)	15 D<br>51 H	21 (18-27)<br>25 (19-32)	39/65	RH<br>RH	> 90 dB	preL, SL	NS	most genetics and meningitidis	GM: no diff<br>WM: ↓ L HG
Shiohama T, 2019	WB	region and surface-based analysis (gyrification index, CT, surface, volume)	15 D<br>36 H	6,7 ± 5,2<br>6,7 ± 5	16/32	NS<br>NS	> 56 dB	preL, NS	yes (convention al or CI)	NS	GM: ↓ CT, surface areas and cortical volume of L MOG and L IOG<br>WM: no diff
Smith K, 2011	HG, WB	manual volumetry,	15 D<br>26 H	14 months ± 3<br>12 months ± 2,6	26/16	/<br>/	> 70 dB (15), > 50 dB (1)	preL, /	NS	NS	GM: ↑ ant HG<br>WM: ↓ ant HG, bilat STG, bilat cerebellum, R MTG, L temp, L post parietal, L occ
Smittenaar C, 2016	V1 (primary visula cortex), defined by fMRI	CT	15 D<br>15 HS	39 ± 10,2<br>38,32 ± 7,9	18/12	RH<br>RH	severe to profound	preL, SL	NS	5 maternal rubella, 3 genetics, 6 unknown	GM: ↓ CT V1<br>WM: /

Note: * and °, same population in different papers; /, not investigated; WB, Whole Brain; VBM, Voxel Based Morphometry; GM, Grey Matter; WM, White Matter; CT, Cortical Thickness; D, Deaf; H, Hearing not signer; HS, Hearing Signers; RH, Right Handed; preL, PreLingual; PostL, PostLingual; NS, Not Specified; Thal, Thalamus; IC, Inferior Colliculi; SC, Superior Colliculi; CC, Corpus Callosum; D, Deaf; H, Hearing; SL, Sign Language; STG, Superior Temporal Gyrus; HG, Heschl's gyrus; PT, Planum Temporale; MTG, Middle Temporal Gyrus; ITG, Inferior Temporal Gyrus; SFG, Superior Frontal Gyrus; MFG, Middle Frontal Gyrus; IFG, Inferior Frontal Gyrus; SOG, Superior Occipital Gyrus; MOG, Middle Occipital Gyrus; IOG, Inferior Occipital Gyrus; CS, Calcarine Sulcus; Fus, Fusiform gyrus; Cing, Cingulate gyrus; preC, precentral gyrus; postC, postcentral gyrus; ↓, decrease (of volume when not specified); ↑, increase (of volume when not specified); R, Right; L, Left; CI, Cochlear Implant; Rw as, Rightward asymetry; Lw as, Leftward asymetry; CMV, Cytomegalovirus
